# Testing the applicability and performance of Auto ML for potential applications in diagnostic neuroradiology

**DOI:** 10.1038/s41598-022-18028-8

**Published:** 2022-08-11

**Authors:** Manfred Musigmann, Burak Han Akkurt, Hermann Krähling, Nabila Gala Nacul, Luca Remonda, Thomas Sartoretti, Dylan Henssen, Benjamin Brokinkel, Walter Stummer, Walter Heindel, Manoj Mannil

**Affiliations:** 1 University Clinic for Radiology, Westfälische Wilhelms-University Muenster and University Hospital Münster, Albert-Schweitzer-Campus 1, 48149 Muenster, Germany; 2 Institute of Neuroradiology, Kantonsspital Aarau, Aarau, Switzerland; 3 Faculty of Medicine, University of Bern, Bern, Switzerland; 4 Faculty of Medicine, University of Zürich, Zürich, Switzerland; 5 Department of Medical Imaging, Radboud University Medical Center, Nijmegen, The Netherlands; 6 Department of Neurosurgery, Westfälische Wilhelms-University Muenster and University Hospital Muenster, Albert-Schweitzer-Campus 1, 48149 Muenster, Germany

**Keywords:** Neurology, Oncology, Outcomes research

## Abstract

To investigate the applicability and performance of automated machine learning (AutoML) for potential applications in diagnostic neuroradiology. In the medical sector, there is a rapidly growing demand for machine learning methods, but only a limited number of corresponding experts. The comparatively simple handling of AutoML should enable even non-experts to develop adequate machine learning models with manageable effort. We aim to investigate the feasibility as well as the advantages and disadvantages of developing AutoML models compared to developing conventional machine learning models. We discuss the results in relation to a concrete example of a medical prediction application. In this retrospective IRB-approved study, a cohort of 107 patients who underwent gross total meningioma resection and a second cohort of 31 patients who underwent subtotal resection were included. Image segmentation of the contrast enhancing parts of the tumor was performed semi-automatically using the open-source software platform 3D Slicer. A total of 107 radiomic features were extracted by hand-delineated regions of interest from the pre-treatment MRI images of each patient. Within the AutoML approach, 20 different machine learning algorithms were trained and tested simultaneously. For comparison, a neural network and different conventional machine learning algorithms were trained and tested. With respect to the exemplary medical prediction application used in this study to evaluate the performance of Auto ML, namely the pre-treatment prediction of the achievable resection status of meningioma, AutoML achieved remarkable performance nearly equivalent to that of a feed-forward neural network with a single hidden layer. However, in the clinical case study considered here, logistic regression outperformed the AutoML algorithm. Using independent test data, we observed the following classification results (AutoML/neural network/logistic regression): mean area under the curve = 0.849/0.879/0.900, mean accuracy = 0.821/0.839/0.881, mean kappa = 0.465/0.491/0.644, mean sensitivity = 0.578/0.577/0.692 and mean specificity = 0.891/0.914/0.936. The results obtained with AutoML are therefore very promising. However, the AutoML models in our study did not yet show the corresponding performance of the best models obtained with conventional machine learning methods. While AutoML may facilitate and simplify the task of training and testing machine learning algorithms as applied in the field of neuroradiology and medical imaging, a considerable amount of expert knowledge may still be needed to develop models with the highest possible discriminatory power for diagnostic neuroradiology.

## Introduction

Artificial intelligence (AI), a branch of computer science that attempts to imitate human thinking and learning using suitable algorithms is nowadays a valuable aid and tool in numerous issues of medical diagnostics. Machine learning (ML), in turn, is an important subcategory of AI. In general, machine learning denotes the use and development of computer systems that are able to learn autonomously using appropriate algorithms to analyze patterns in data and draw appropriate conclusions. Usually, a distinction is made between “supervised learning”, “unsupervised learning” and “reinforcement learning”. Supervised learning algorithms are first trained with known data and results (i.e. with labeled data). Using the previously learned logic, the algorithms are then able to classify unknown data accordingly. An important subcategory of supervised learning is “deep learning”. These algorithms use artificial neural networks that often contain many layers and are usually trained with a large set of labeled data. In contrast, with unsupervised learning, the algorithms independently search for previously unknown patterns in unlabeled data. Finally, reinforcement learning is an iterative approach in which algorithms learn independently by means of reward and punishment (i.e. by trial and error).

For several years, the number of machine learning publications in medical research has steadily increased^1^. However, a considerable amount of expert knowledge is required to apply these methods appropriately. Importantly, many time-consuming and difficult steps are required to allow for a suitable use of supervised machine learning algorithms, such as the appropriate separation of data into training and test data, feature preparation, feature preselection, the final multivariate feature selection, the optimization of hyperparameters included in the models, and the final model construction and selection. Furthermore, underfitting and overfitting should be avoided in model design and models should be optimized in terms of stability so that model performance is largely independent of the selected data sample. The final goal is to design a good model that has high and similar discriminatory power both with independent test data as well as with the training data as implemented for initial model development. All these individual items are challenging and require a high level of professional experience.

Given these challenges, researchers have sought ways to facilitate and simplify these tasks to promote the further distribution of machine learning in business and science. Therein, automated machine learning (AutoML) has emerged as a means of simplifying and automating these steps and thus make machine learning accessible to non-experts. Many AutoML algorithms are already highly automated. In principle, complete models can be developed and tested comparatively easily. Starting with the raw data, many AutoML algorithms involve the automated splitting of the data into training and test data, the preparation of the variables, the pre-selection and selection of suitable features, the determination of the hyperparameters, the parallel computation of a whole set of different models, and finally the independent selection of the best of these models. With just a few lines of computer code, AutoML can be used to test numerous machine learning algorithms simultaneously.

This high level of automation should also enable non-experts to develop machine learning models. However, automation also offers numerous potential benefits for experts. For example, it is conceivable to use automated machine learning to get a quick overview of which model algorithms might be promising for detailed further analysis.

Conventional machine learning and deep learning algorithms have many possible applications in diagnostic neuroradiology. In recent years, for example, a machine learning predictive model has been developed for the diagnosis of brain tumors based on routine blood tests^2^. Such algorithms can also be used for an automated detection and segmentation of meningiomas^3^ and a preoperative classification of WHO grade of meningiomas and gliomas^4–6^.

Recently, AutoML has also been increasingly used in the areas of diagnostics, medical modeling, and imaging. Exemplarily, AutoML has shown promise for the discrimination of severe from non-severe COVID-19 patients, COVID-19 patients from patients with another acute respiratory illness, and COVID-19 patients from virus-free individuals^7^. Moreover, AutoML algorithms have also been used to predict the chances of surviving a SARS-CoV-2 infection^8^.

Many studies have already been published on conventional machine learning, but only comparatively few on automated machine learning. Despite the demonstrated need, there are still few efforts to use these techniques in the health sector^9,10^. In this study, we aim to analyze the potential of AutoML for neuroradiology using an exemplary but important medical prediction application. At least since Simpson's work published in 1957, it has been known that extent of meningioma resection is an important factor to predict the risk of tumor recurrence^11,12^. Therefore, early knowledge of the achievable postoperative resection status is of great importance for further therapy planning. To test the applicability of AutoML for potential applications in neuroradiology, we attempt to predict possible gross total resections (GTRs) of meningiomas from pre-treatment MR-images using radiomics and AutoML. Therein, we distinguish cases with possible gross total resection from cases where only subtotal resection (STR) can be performed.

We assess the performance of AutoML for the task at hand, compare AutoML systematically with conventional machine learning algorithms and discuss the respective advantages and disadvantages. Our aim is to obtain an assessment of whether non-experts can already use AutoML to develop similarly good models for applications in diagnostic neuroradiology as technically skilled developers of conventional machine learning models.

## Materials and methods

This single center study was performed in compliance with the Declaration of Helsinki and approved by the local ethics committee (Ärztekammer Westfalen Lippe and University of Münster, 2021-596-f-S). Due to the retrospective nature of the study, written informed consent was waived by the Ärztekammer Westfalen Lippe and University of Münster.

We retrospectively searched our database for patients diagnosed with meningioma followed by resection between February 2015 and July 2018. 167 patients were initially screened. Our final cohort included 100 female and 38 male. The 29 patients excluded had (1) missing or non-diagnostic pre-treatment cerebral magnetic resonance imaging, (2) insufficient diagnostic imaging quality, (3) incomplete clinical data, (4) inconsistent histopathology or (5) insufficient follow-up examinations. Gross total resection of the meningioma was performed in 107 cases and subtotal resection in the remaining 31 cases. The definition of GTR/STR was based on census between the responsible neurosurgeon and postoperative MR imaging.

**Figure 1:**
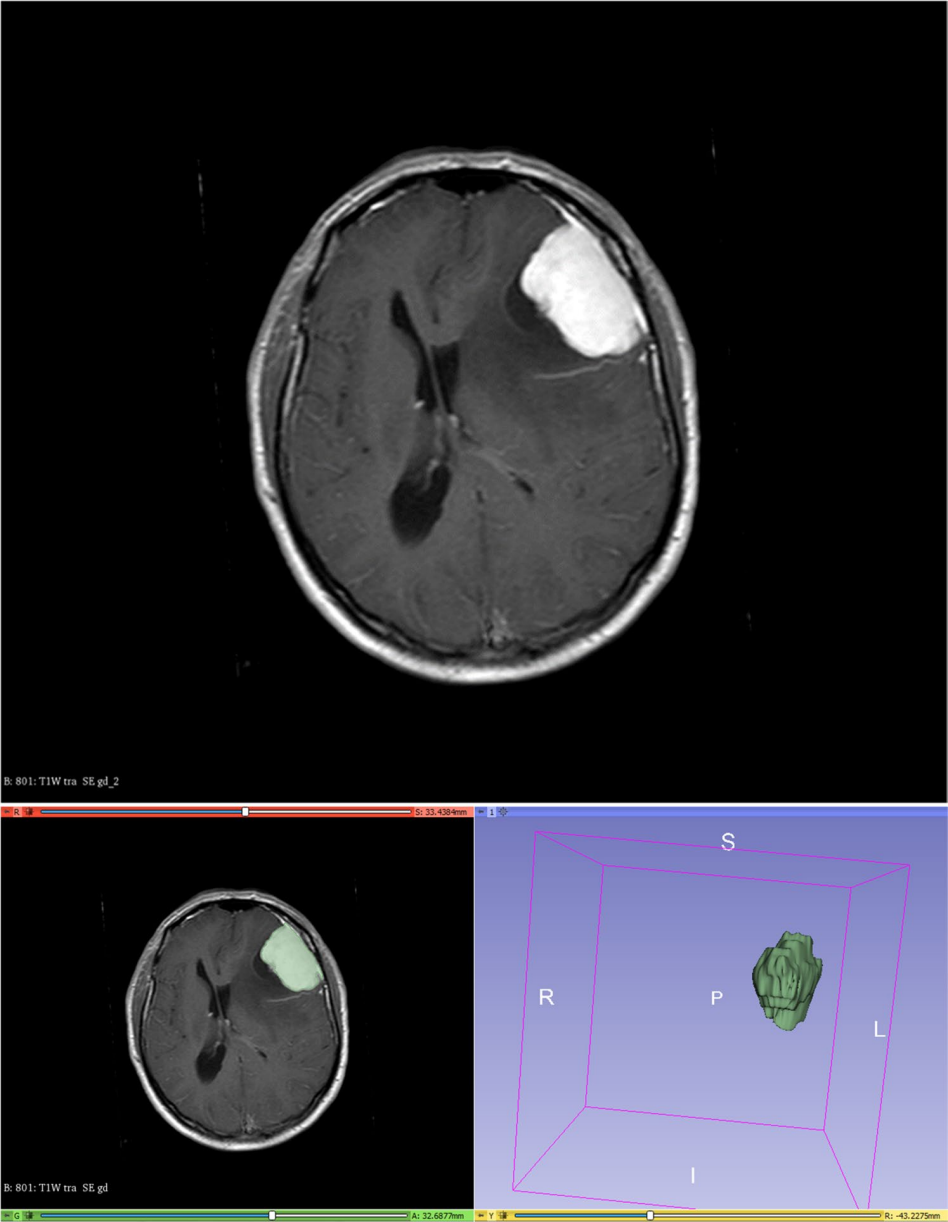
Convexity meningioma of the left hemisphere (above); semi-automatic segmentation with 3D Slicer (below).

Segmentation of the contrast-enhancing parts of the tumor was performed semi-automatically using the open-source software platform 3D Slicer (version 4.10, www.slicer.org). As an example, a convexity meningioma is shown in Fig. 1. The figure shows the semi-automatic segmentation with 3D Slicer.

A total of 107 radiomic features were extracted from the MRI images of each patient by hand-delineated regions of interest (ROI). These 107 radiometric features can be assigned to seven different feature classes: (1) first-order statistics (18 features), (2) shape-based features (14 features), (3) gray-level co-occurrence matrix (24 features), (4) gray-level run length matrix (16 features), (5) gray-level size zone matrix (16 features), (6) adjacent gray-level difference matrix (5 features), and (7) gray-level dependency matrix (14 features). In addition, our database contained further factors, such as gender and age, the Karnofsky Performance Scale Index (KPI), the location of the meningioma in the brain, the shape and the subtype of the tumor and the distinction between a first diagnosis of the tumor and a relapse. Most categorical features were used in binary form. All features were z-score transformed and then subjected to a 95% correlation filter to account for redundancy between the features.

For our analyses, we used conventional machine learning algorithms, a neural network, and AutoML. In R, there are already several open source AutoML frameworks, such as H2O AutoML, the automl package available on CRAN, Remix AutoML and AutoXGboost. They differ, among other things, in the selection and number of machine learning algorithms available. We used the H2O AutoML algorithm for our analyses. This algorithm is characterized on the one hand by its comparatively high speed and on the other hand by the special feature that the number of models to be trained and the maximum time for training the models can be limited. The H2O AutoML algorithm simultaneously optimizes models belonging to different model classes: GBMs (Gradient Boosting Machines) and XGBoost GBMs, GLMs (Generalized Linear Models), DNNs (Deep Neural Networks), an XRT (Extremely Randomized Trees) and a DRF (Distributed Random Forest). Some of these model classes contain multiple models. In addition, H2O AutoML uses combinations of these base algorithms, called “Stacked Ensemble Models (SEMs)”. On the one hand, this methodology can lead to a further improvement in the forecasting quality of the models, but on the other hand it also harbours the danger that the corresponding models then have a black-box character due to their even greater complexity. The functionality of H2O AutoML is extremely diverse and can only be hinted at here. Detailed further information can be found in the H2O AutoML documentation^13^. For our analyses, we used all the algorithms listed except XGBoost GBMs and the SEMs (stacked ensembles).

To enable the most accurate comparison of AutoML with the various conventional machine learning algorithms, we have, as will be described, placed great emphasis in our analyses on eliminating random influences on the results as much as possible. For example, each model is fully developed 100 times with new training data sets and then tested 100 times with associated different independent test data sets. For all comparisons between the various algorithms, the values calculated with the 100 sets of independent test data, averaged over these 100 runs, are used. In addition, we use a technique that results in final models with as few features as possible to also avoid overfitting the models as much as possible.

### Statistical analysis

Statistical analysis was performed with R software (version 3.5.3). Numerous packages are available in R for both conventional machine learning algorithms and automated machine learning. We used the “H2O” package for the automated machine learning/AutoML and the “caret” package for the feed-forward neural network with a single hidden layer (“method = nnet”) and the conventional machine learning algorithms.

Our final cohort of 138 patients was randomly divided into training data and independent test data. For data partitioning, a stratified ratio of approximately 80:20 was used with a balanced distribution of resection status (GTR/STR) and gender (female/male) between the two samples (Table 1). The training data of each split contained exactly 111 patients (80.4% of the complete final cohort of 138 patients) and the test data contained the remaining 27 patients (19.6% of the complete final cohort). Construction of the models and determination of the hyperparameters included in the models were performed with the training data (including validation data). The performance of the models was subsequently determined with the test data (i.e. using unknown/independent data). To avoid random effects related to the partitioning of the data, this procedure was repeated 100 times for each individual model. For each of these 100 data partitions, the assignment of the 138 patients to the training data and the test data changed. However, the number in each of the two groups (training data: 111 patients, test data: 27) was kept constant. We calculated all performance values as the average of these c = 100 cycles. In order to compare the results obtained with the different machine learning algorithms as accurately as possible, each model was developed and tested with the same 100 training and test samples. To achieve this, we used for each model the same series of 100 different seeds “s” for the 100 data splits.

**Table 1:** Clinical and demographic data.

	Training data	Independent test data
Number	111	27
**Gross total resection or subtotal resection (in %)**		
GTR	77.48	77.78
STR	22.52	22.22
Mean age (years)	58.80	59.12
**Gender (in %)**		
Male	27.93	25.93
Female	72.07	74.07

**Figure 2:**
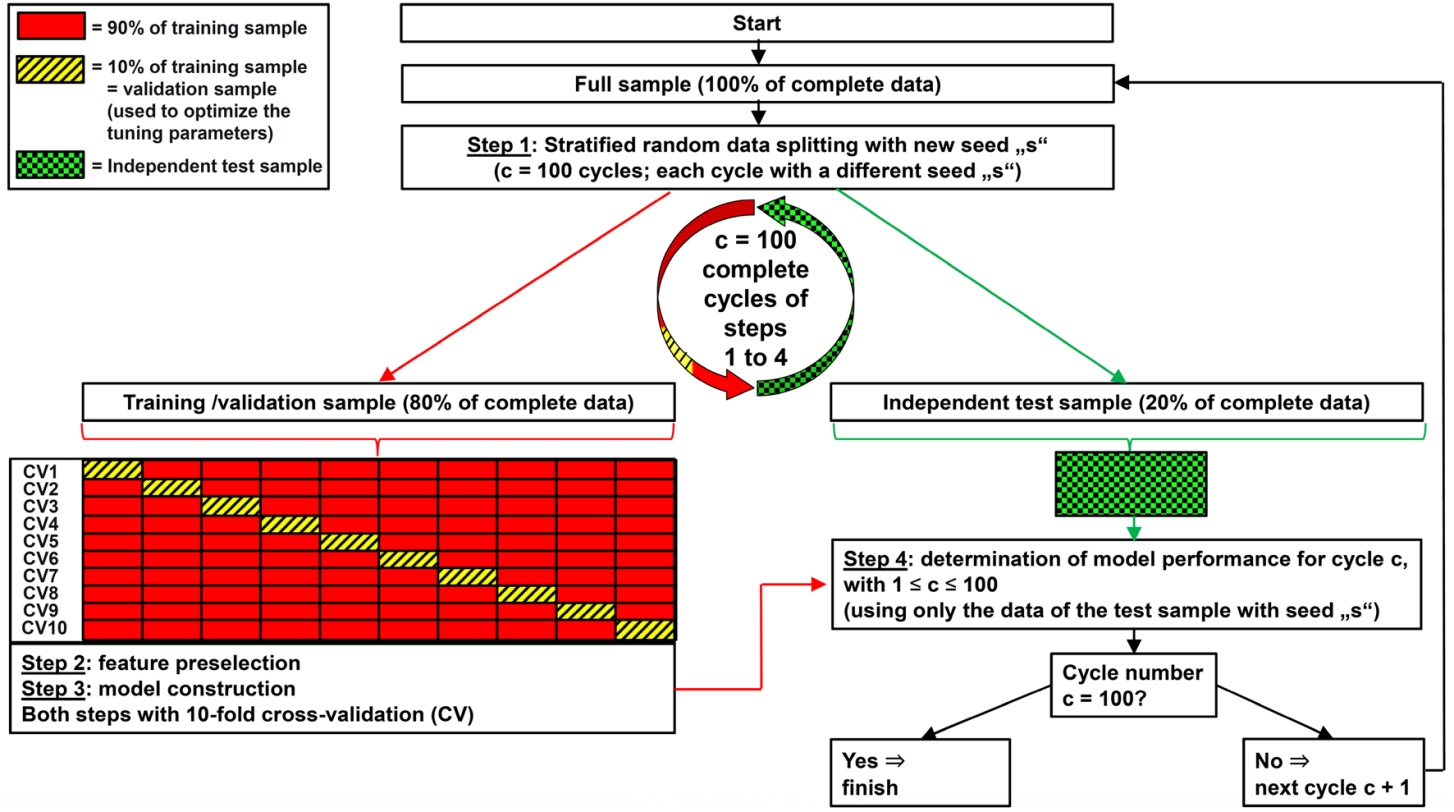
Development and test of a model with 100 repetitions (c = 100 cycles), fixed number of features und a fixed machine learning algorithm used for feature preselection and for the subsequent model estimation.

A four-step approach was used to construct and test each model (see Fig. 2). In the first step, as already explained, the full data set was divided into training and test data. In the second step, the feature preselection of the most important features was performed. We used the “varImp” function in R to identify these most important (most discriminant) features. This function determines the additional performance of each feature included in a model. We also determined the univariate discriminatory power of the features and their statistical significance. We performed the chi-square test (Fisher’s exact test) for binary and categorical features. All continuous features were first analyzed using the Shapiro–Wilk normality test. In case of equal variance in the two groups (GTR/STR), normally distributed features were further analyzed with Student's t-test and in case of unequal variance with Welch's test. Finally, non-normally distributed continuous variables were analyzed using the Wilcoxon test (Mann–Whitney*-*U-Test). Table 2 shows all features with *p*-values < 0.1. In the third step, the models containing the features identified in the second step were estimated. Finally, in the fourth step, the models were tested using the unknown/independent test sample. This four-step approach was completely repeated 100 times for each model. All performance values were calculated as means of these 100 runs.

**Table 2:** Univariate results.

Variable	GRT (n = 107)	STR (n = 31)	*p*-value
^1^tumor location = skull base	38 (35.5%)	26 (83.9%)	0.00001*
^1^tumor location = convexity	46 (43.0%)	0 (0.0%)	0.00002*
^1^tumor shape = irregular	31 (29.0%)	21 (67.7%)	0.00021*
^1^fd_vs_re = rezidiv	6 (5.6%)	9 (29.0%)	0.00077*
^1^Tumor location = falx	17 (15.9%)	0 (0.0%)	0.03942*
^2^orig.glszm.Smallareaemphasis	0.578 (0.388, 0.750)	0.727 (0.529, 0.813)	0.04842*
^1^KPI (50/60/70/80/0/100)	3/0/5/30/50/19	1/2/2/13/11/2	0.04638*
^2^orig.shape.Elongation	1.000 (1.000, 1.000)	1.000 (0.791, 1.000)	0.05904
^2^orig.shape.MajorAxisLength	3.068 (2.000, 3.266)	3.266 (2.530, 3.266)	0.06798
^2^orig.glszm.ZoneEntropy	1,585 (1.500, 2.322)	2.000 (1.585, 2.322)	0.09144

^1^Binary and categorical features: number n (in %), ^2^continuous variables: median (interquartile range).

*p-value < 0.05 (assumed to be statistically significant).

First, the feature preselection (step 2) and subsequent model construction (step 3) was performed with AutoML. Then, to compare the obtained results, we trained and tested different conventional machine learning algorithms including a neural network. Specifically, we used the following algorithms: Logistic regression, Lasso regression, Ridge regression, Gradient Boosting Machine (GBM), Random forest, Bagged trees, LDA (Linear Discriminant Analysis), Naive Bayes and finally a feed-forward neural network with a single hidden layer.

Our models were created using an increasing number of the most important features identified in each second step. Initially, each model contained only the most important feature, followed by a model with the two most important features, followed by a model with the three most important features, and so on. For each machine learning algorithm, the model with the highest performance using the independent test data was selected as the final model. This step-by-step approach determines the final numbers of features included in each model. The approach described here was performed independently for AutoML, the neural network and each of the conventional machine learning algorithms listed above. The aim of this approach with an increasing number of features is to avoid both underfitting and overfitting. Our objective is to find models with high discriminatory power and at the same time as few features as possible.

The model optimization was performed for all models by maximizing of the Area Under the Curve (AUC) of the Receiver Operator Characteristic (ROC). The predictive power of each model was analyzed using AUC, accuracy, Cohen’s kappa, sensitivity and specificity. Since AutoML optimizes numerous models at the same time, one (the best) of these models had to be selected. We tried two metrics to determine the best AutoML model. The models were selected according to the highest AUC value on the one hand and the lowest (best) *Log Loss* value on the other. When using H2O AutoML, the maximum training time for the models can be limited. We have used a maximum training time of 100 s (for each cycle). We have also tested significantly longer maximum training times. However, these did not further improve the results obtained with AutoML.

For both, feature preselection and subsequent model constructions only the training data were used. This applies to AutoML as well as to the neural network and the conventional machine learning algorithms. The hyperparameters included in the models were determined using a grid search 10-fold cross-validation. Therefore, we divided the training data 10 times into groups with 90% and 10% of the training data (the data of the 10% groups are called validation data). This technique ensures that the 10%-subgroups used to optimize the hyperparameters do not overlap. It is a methodology often used to obtain robust results with small datasets and to avoid overfitting. To determine the hyperparameters, models for many possible different values of the hyperparameters and their combinations (hyperparameter sets) were estimated using 90% of the training data, i.e. cross-validation by grid search. The performance of these models was then determined using the first remaining 10% of the training data (the first group of validation data). This procedure was repeated ten times for each set of hyperparameters, each time using a different 90% of the training data for the actual training and the remaining 10% as validation data (10-fold cross-validation). Finally, the set of hyperparameters that performed best on average with the validation data was selected.

To summarize, each of our models with a fixed number of features and a fixed machine learning algorithm has been fully constructed, estimated and tested 100 times. For this purpose, first the stratified splitting of the full dataset into training data (80% of data) and test data (20% of data) described above was repeated c = 100 times (step 1 in Fig. 2) using different seeds “s”. This means that for each model we used 100 different training samples and 100 different test samples with independent data. Variable preselection was then performed with each of these training samples (step 2), then each of the 100 models was estimated using the respective training sample number “c” (step 3), and finally each final model number “c” was tested with the respective independent test sample number “c” (step 4). The complete process for developing and testing a single model with a fixed number of features and a fixed machine learning algorithm is shown in Fig. 2. We performed this complex approach with 100 cycles/repetitions to eliminate any random effects as far as possible.

## Results

### Determining the best metric for model selection with AutoML

**Figure 3:**
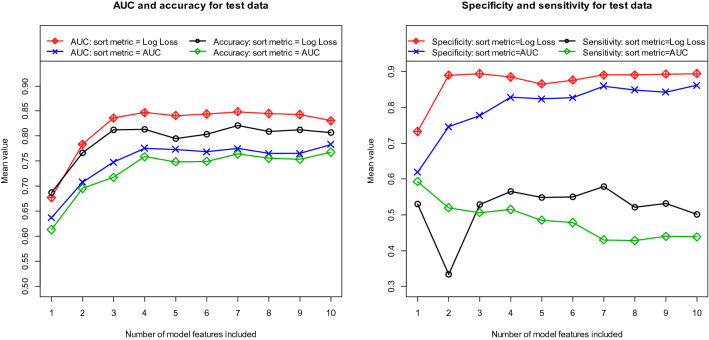
Performance results for models built with Automated Machine Learning (AutoML). Left figure: Area Under the Curve (AUC) and accuracy. Right figure: specificity and sensitivity. All values calculated as means of 100 repetitions (100 cycles) using independent test data. Sort metric used to specify the best model in each cycle: *Log Loss* (red and black lines) and AUC (blue and green lines).

We started our analyses by determining the best model selection metric for the AutoML algorithm. Two metrics were used to select the best model in each cycle, as described earlier. First, we determined the best model with respect to the AUC metric and, second, to the *Log Loss* metric. Figure 3 shows the performance results for AUC and accuracy in the left part of the figure and the results for specificity and sensitivity in the right part of the figure. For the clinical case study considered here, the *Log Loss* metric leads to significantly better results than the AUC metric. From the fourth variable onwards, adding more features does not significantly increase the discriminatory power of the model. Using the *Log Loss* metric to predict the best model, the four-feature model yields a mean AUC, mean accuracy, mean sensitivity, and mean specificity of 0.847, 0.814, 0.565, and 0.885, respectively. Since the *Log Loss* metric led to significantly better results than the AUC metric, the *Log Loss* metric was generally used for all further analyses to select the best model.

### Classification results for AutoML, the neural network and the conventional models

**Figure 4:**
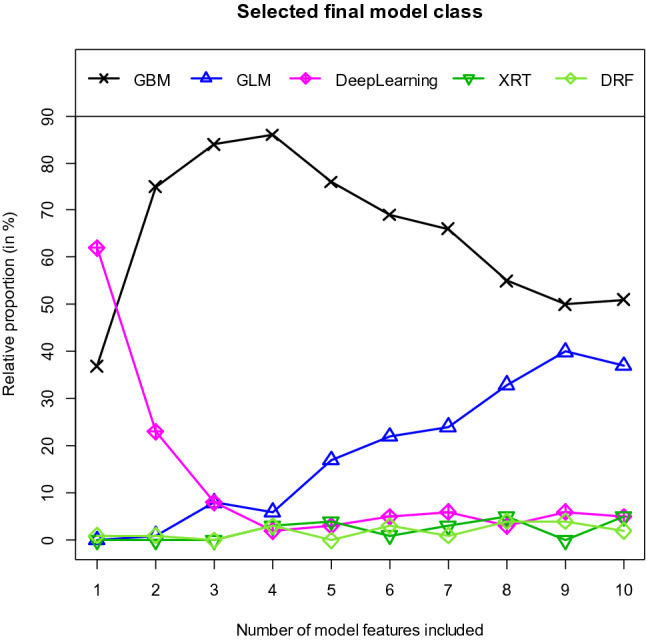
Selected final model class. *GBM*  Gradient Boosting Machine, *GLM *Generalized Linear Model, DeepLearning = Fully-connected multi-layer artificial neural network, *XRT *Extremely Randomized Trees, *DRF *Distributed Random Forest.

As explained earlier, we estimated each model with a fixed number of features included in the model 100 times. Figure 4 shows the frequencies of the model classes selected by AutoML. The most common choice (62 of 100 cases) for single-feature models is a neural network. Most of the models with more than one feature are GBM models. As the number of variables included in the models increases, the importance of GBM models decreases and the importance of GLM models increases. It is interesting to note that the models belonging to the class of decision trees (XRT and DRF) are only rarely selected.

**Figure 5:**
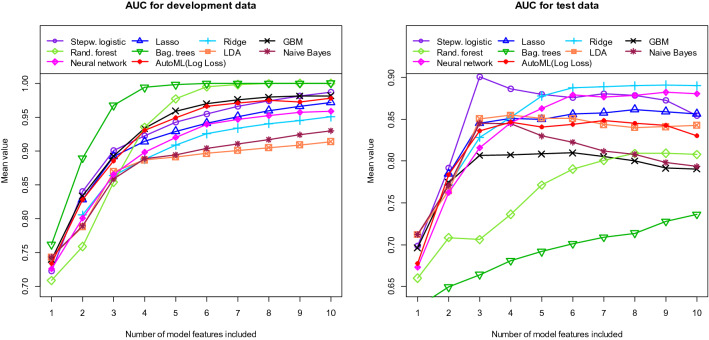
Area Under the Curve (AUC) for AutoML (red lines), neural network (pink lines) and conventional machine learning models (other lines). Left figure: training data. Right figure: independent test data. All values calculated as means of 100 repetitions (100 cycles).

**Figure 6:**
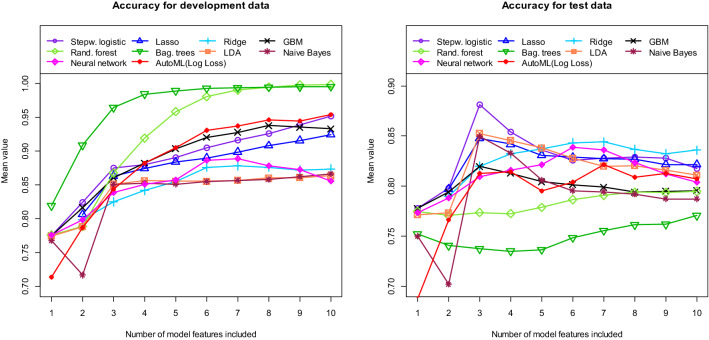
Accuracy for AutoML (red lines), neural network (pink lines) and conventional machine learning models (other lines). Left figure: training data. Right figure: independent test data. All values calculated as means of 100 repetitions (100 cycles).

We then also trained and tested a neural network and conventional machine learning models. All tested models belong to the model classes included in AutoML. The left part of Fig. 5 shows the AUC values obtained with the training data and the right part shows the corresponding results obtained with the independent test data. Some of the curves obtained with the test data already show a decreasing trend from a certain number of variables (for example the curve of the logistic regression with more than 3 variables). From these points onwards there is an overfitting in the respective models. Figure 6 shows the results for the accuracy. Using the training data, AutoML performs well compared to the neural network and most of the conventional models. However, when using the test data, the performance of AutoML is only in the middle range.

When using the training data, the AutoML algorithm provides very similar results to conventional GBM models. As shown in Fig. 4, the AutoML algorithm also selects a GBM model in most cases. This explains the similarity of the results of these two algorithms.

In accordance with the AutoML algorithm, the performance obtained with the independent test data does not increase further for most of the conventional models when using more than three or four features. For all three methodologies, the AutoML algorithm, the neural network and the conventional machine learning algorithms, the tumor shape (irregular or regular) and its location in the brain are by far the most important factors. Thus, the models all use very similar variables. Nevertheless, very different performances are obtained with the different algorithms.

Using the training data, the performance of the models increases with model complexity. Models with more hyperparameters such as bagged trees, random forest or GBM as well as models containing more variables lead to higher discriminatory power values. However, the opposite is true when using the independent test data. Here, the comparatively simple models of the GLM class, such as a logistic regression, yield the best results. In addition, the use of more than three variables does not result in a significantly higher discriminatory power for most models. These findings become even more obvious when we calculate the Relative Loss of Performance (RLP). We define the RLP as follows:

$$ RLP = {{\left( {Performance\;with\;test\;data{-}performance\;with\;training\;data} \right)} \mathord{\left/ {\vphantom {{\left( {Performance\;with\;test\;data{-}performance\;with\;training\;data} \right)} {performance\;with\;training\;data}}} \right. \kern-\nulldelimiterspace} {performance\;with\;training\;data}} $$

**Figure 7:**
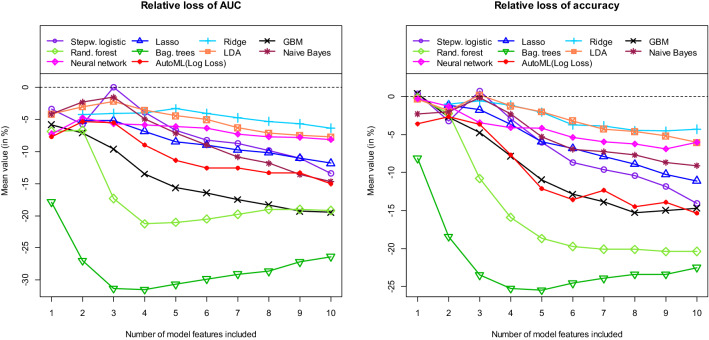
Relative Loss of Performance (RLP) for AutoML (red lines), neural network (pink lines) and conventional machine learning models (other lines). Left figure: AUC. Right figure: accuracy. All values calculated as means of 100 repetitions (100 cycles).

In Fig. 7, the RLPs are shown for AUC and accuracy. The relatively simple logistic regression model with only three features has almost no loss of performance when switching from training to test data. Therefore, this model shows an extremely robust prediction quality. Based on the training data, most GBM models and some of the neural nets are slightly stronger than the logistic regression. However, these models, which are used extensively by the AutoML algorithm, have high RLPs. This in turn leads to a somewhat limited performance of the AutoML algorithm in our clinical case study when using the independent test data. It is remarkable that AutoML selects almost no models of the classes XRT and DRF. In relation to our case study, this is a strength of the AutoML algorithm. As our results for the bagged trees and random forest models show, corresponding conventional models belonging to this model class exhibit high RLPs and are therefore rather unstable.

### Comparison of the methodologies

Finally, we compare the performance obtained with the best AutoML model with the results obtained with the neural network as well as with the best of the conventional models. From each set of models with a different number of features, the model with the highest discriminatory power (AUC) with respect to the independent test data is selected. This method prevents both underfitting and overfitting. Using the independent test data, the highest performance of the AutoML algorithm is obtained with the seven-feature model and for the neural network with six features. The best results for the conventional machine learning models are obtained with the logistic regression model including only three features. It should be noted, that the two AutoML models with four and with seven features have a very similar performance. Thus, the number of variables required in the AutoML model and the logistic model are comparable. However, the performance of the neural networks with three and four features is significantly lower compared to the neural network with six variables (compare Figs. 5 and 6). Table 3 summarizes the classification results for these three best models (AutoML, neural network and logistic regression). The table also contains the results for the AutoML model with 4 variables, which are very similar to the results of the model with 7 variables. The values in the brackets indicate the 95% confidence interval (CI). The AutoML algorithm yields good discriminatory power. The performance of the AutoML algorithm is very close to the results achieved with the neural network. This finding indicates a great potential of AutoML. However, in the case study considered here, simple logistic regression yields even better discriminatory power with respect to all five performance measures considered. The difference is particularly evident for sensitivity and Cohen’s kappa. In our study, sensitivity evaluates how frequently cases with subtotal resection (STR cases) are correctly predicted. Here, the logistic regression performs significantly better than both neural network and AutoML. Specificity, on the other hand, evaluates how often the GTR cases are correctly predicted.

**Table 3:** Classification results for AutoML (using 4 and 7 features), neural network (using 6 features) and logistic regression (using 3 features).

	AutoML (4 features)	AutoML (7 features)	Neural network	Logistic regression
AUC	0.847 [0.642, 0.975]	0.849 [0.675, 0.978]	0.879 [0.716, 0.984]	0.900 [0.786, 0.976]
Accuracy	0.814 [0.667, 0.926]	0.821 [0.704, 0.926]	0.839 [0.704, 0.944]	0.881 [0.778, 0.963]
Kappa	0.450 [− 0.013, 0.786]	0.465 [0.087, 0.757]	0.491 [0.000, 0.847]	0.644 [0.348, 0.899]
Sensitivity	0.565 [0.000, 1.000]	0.578 [0.167, 1.000]	0.577 [0.000, 1.000]	0.692 [0.333, 1.000]
Specificity	0.885 [0.619, 1.000]	0.891 [0.737, 1.000]	0.914 [0.786, 1.000]	0.936 [0.857, 1.000]

All values calculated as means of 100 repetitions (100 cycles) using independent test data. Values in brackets: 95% confidence interval.

Lastly, it should be noted that we also tested other metrics besides the AUC metric, such as the area under the precision-recall curve (AUPRC), a metric often used for imbalanced data. In our case, this did not lead to a significantly higher discriminatory power of the models. However, one of the main aims of our study was to compare AutoML with a neural network and conventional machine learning algorithms anyway. For this purpose, the choice of metric used has somewhat less importance. To be able to compare the results fairly, it is particularly important that the same metric was used for all algorithms, as we did in our study.

## Discussion

In this study, we analyzed the performance of AutoML in relation to a neuroradiology case study, specifically for predicting the achievable resection status of meningiomas. We compared the performance of AutoML with corresponding results obtained with a neural network and conventional machine learning algorithms. Based on our training data, we found good discriminatory power of the AutoML algorithm. However, with respect to the independent test data, the relative simple logistic regression yields a slightly higher discriminatory power, especially in terms of sensitivity and kappa.

One reason why the more complex models, i.e. the AutoML algorithm and the neural network, did not perform better than the logistic regression may be that only a few of the features included in our database had a high univariate performance in discriminating STR and GTR cases. Table 2 summarizes all features with a univariate *p*-value < 0.1. If we consider *p*-values < 0.05 as statistically significant, then only the different possible locations of the tumor in the brain, the shape of the tumor (regular or irregular), the distinction between a first diagnosed and a recurrent tumor, the Karnofsky Performance Scale Index (KPI) and finally an additional continuous feature (orig.glszm.SmallAreaEmphasis) have significant discriminatory power. However, variables that do not have high univariate discriminatory power can also contribute to the performance of a multivariable model. Accordingly, we did not exclude these variables in our analyses. As a rule, however, the discriminatory power of a multivariate model can be further increased with variables that also show significant discriminatory power univariately. The most important three to four variables are the same or at least very similar in all the models we tested. These are the features that also have univariate significant discriminatory power. Additional variables did not further increase the discriminatory power of the models when using the independent test data. Therefore, the possibilities of achieving even higher discriminatory power with more complex algorithms than logistic regression such as AutoML are certainly limited to a certain extent. To achieve high discriminatory power with an AutoML algorithm, it is important to find a suitable combination of feature selection and processing, model selection and tuning of hyperparameters. Due to the many factors involved, this task can quickly become very complex and computationally intensive. Combinations of the individual steps mentioned are also referred to as "pipelines". "Pipeline optimizers" are systems that support the automation of multiple machine learning steps. Such optimization algorithms have a high potential to improve the discriminatory power of automated machine learning. Two important pipeline optimization algorithms are TPOT (Tree-based Pipeline Optimization Tool)^14^ and Auto-Sklearn^15^. However, such algorithms are especially beneficial for complex problems with many data sets. This is precisely not the case in our study. Nevertheless, logistic regression outperforms the much more flexible AutoML algorithm in our clinical case study. In addition, logistic regression models are often very easy to interpret. For example, our logistic model with only three variables establishes an easy-to-understand relationship between the location of a tumor in the brain, its shape and the diagnostic question of whether this tumor can be completely resected or not. On the other hand, the GBM models frequently selected by the AutoML algorithm and especially the so-called stacked models (SEMs) we excluded are much more difficult to interpret. These models still have a certain black-box characteristic.

Neural networks also still often have a certain black-box characteristic. Another disadvantage of neural networks is that large amounts of data are often required to train the algorithm in order to obtain generalizable results. This can be associated with correspondingly long computing times. However, in many cases neural networks succeed in finding good solutions even for complex and strongly nonlinear problems.

Compared to conventional machine learning algorithms and neural networks, AutoML offers numerous advantages. The first thing to mention here is the comparatively simple development of even complex models. Many parameters, such as the algorithms themselves to be tested or the simulation time can be specified. Even neural networks can be included. AutoML provides a comparatively easy way to determine what kinds of algorithms might be particularly discriminating and should be analyzed in more detail. Automated machine learning is a very promising tool for medical research and diagnostics. Waring et al. provide a good overview of the basic methodology, state-of-the-art and possibilities of automated machine learning in healthcare^9^. Initial publications demonstrate the high potential of automated machine learning in medical research. Karaglani et al. used automated machine learning to produce predictive biosignatures that provide opportunities for minimally invasive blood-based diagnostic tests for Alzheimer's disease^16^. Ou et al. compared an AutoML algorithm with multivariate regression and a random forest model for predicting intracranial aneurysm treatment outcomes^17^. According to our results, the AutoML algorithm performed better than the conventional random forest model. However, the multivariate regression showed the worst performance of the three models compared in their study. Touma et al. have even developed a completely code-free AutoML model with very high accuracy for classifying cataract surgery phases from videos^18^. These and many other studies demonstrate the great potential that already exists with current AutoML algorithms. However, our study has also shown that the use of AutoML specifically for applications in diagnostic neuroradiology does not yet always lead to equally good or even better results compared to conventional machine learning algorithms. This would be an important prerequisite for a corresponding clinical application of AutoML. With the expected further improvement of the algorithms in the near future, this could certainly become achievable. Before that, however, further corresponding prospective clinical studies are needed.

## Conclusion

There is currently a great demand to further simplify the application of machine learning algorithms, making these algorithms also accessible to non-experts. AutoML is an important and promising step in this direction. As we have seen, it is comparatively easy to develop models with acceptable discriminatory power using AutoML. A simple AutoML model requires only a few lines of computer code. Numerous algorithms can be tested simultaneously. However, the appropriate use of AutoML algorithms still requires a fair amount of expertise. Due to the diversity and complexity of the algorithms included in AutoML, it is comparatively easy to increase the discriminatory power on dependent training data. However, this entails the risk that when independent test samples are used and random effects are excluded, as we did in our study, only an apparent gain in performance is achieved, or at least a significantly lower one. As demonstrated, simpler models can even lead to better results. Our clinical case study shows that conventional machine learning algorithms can still have clear advantages, such as high and very stable model performance and very good interpretability.

## Data Availability

The anonymized data is available upon reasonable request towards the corresponding author.
